# High-performance paper-based biocathode fabricated by screen-printing an improved mesoporous carbon ink and by oriented immobilization of bilirubin oxidase

**DOI:** 10.1038/s41598-022-19052-4

**Published:** 2022-08-27

**Authors:** Noya Loew, Isao Shitanda, Himeka Goto, Hikari Watanabe, Tsutomu Mikawa, Seiya Tsujimura, Masayuki Itagaki

**Affiliations:** 1 Department of Pure and Applied Chemistry, Faculty of Science and Technology, Tokyo University of Science, 2641 Yamazaki, Noda, Chiba 278-8510 Japan; 2 Research Institute for Science and Technology, Tokyo University of Science, 2641 Yamazaki, Noda, Chiba 278-8510 Japan; 3 RIKEN Center for Biosystems Dynamics Research, 1-7-22 Suehirocho, Tsurumiku, Yokohama, Kanagawa 230-0045 Japan; 4 Division of Materials Sciences, Faculty of Pure and Applied Sciences, University of Tsukuba, 1-1-1 Tennodai, Tsukuba, Ibaraki 305-8573 Japan

**Keywords:** Biochemistry, Biocatalysis, Enzymes

## Abstract

In this study, the performance of a paper-based, screen-printed biofuel cell with mesoporous MgO-templated carbon (MgOC) electrodes was improved in two steps. First, a small amount of carboxymethyl cellulose (CMC) was added to the MgOC ink. Next, the cathode was modified with bilirubin prior to immobilizing the bilirubin oxidase (BOD). The CMC increased the accessibility of the mesopores of the MgOC, and subsequently, the performance of both the bioanode and biocathode. CMC also likely increased the stability of the electrodes. The pre-modification with bilirubin improved the orientation of the BOD, which facilitated direct electron transfer. With these two steps, an open circuit potential of 0.65 V, a maximal current density of 1.94 mA cm^−2^, and a maximal power density of 465 μW cm^−2^ was achieved with lactate oxidase as bioanode enzyme and lactate as fuel. This is one of the highest reported performances for a biofuel cell.

## Introduction

Mesoporous carbon materials are one of the most appealing materials for the fabrication of bioelectrochemical devices such as biosensors and biofuel cells^1–3^. These materials combine high conductivity, high surface area, and excellent biocompatibility, they are excellent for electrodes and matrices for enzyme immobilization. Yang et al. reported an increased temperature and pH stability when glucose oxidase was immobilized on ordered mesoporous carbon^4^. Among the different types of mesoporous carbon materials are oxide-templated carbons. The pore size of oxide-templated carbons can be controlled by controlling the size of the oxide template^5–9^. One such oxide-templated carbon is MgO-templated carbon (MgOC), which is commercially available^5,6^. The effect of the pore size of MgOC on the direct electrochemistry has been investigated for D-fructose dehydrogenase^10^ and bilirubin oxidase (BOD)^11,12^. Furthermore, biofuel cells (BFCs) fabricated with MgOC ink-modified carbon cloth had a high-power output of 2 mW cm^−2^^13^ and 4.3 mW cm^−2^^14^ with glucose dehydrogenase (GDH) and lactate oxidase (LOx) as enzymes, respectively.

A MgOC ink is also the first step in fabricating a screen-printed MgOC electrode. The conductive carbon material in screen-printing inks needs to be dispersed evenly under shear stress applied during printing. An uneven dispersion might lead to a partially brittle electrode (where too little binder is present) and/or a partially increased resistance (where too much binder is present). A higher dispersion can also lead to a higher degree of porosity, as clumping becomes less likely. Small quantities of additives can improve the dispersion of ink without interfering with the conductivity, and thus the quality and reproducibility of the printed electrode. However, although biocompatible and sustainable materials, such as carboxymethyl cellulose (CMC), have been used as dispersants for carbon materials^15^, dispersants have not been considered for MgOC inks for screen-printing.

Screen-printed electrodes are promising for the fabrication of wearable biosensors, especially for healthcare applications^16–18^. Wearable biosensors are receiving significant attention in recent years owing to the trend of a more personalized, real-time healthcare management of patients, as well as a more data-driven, closer monitoring of the physical condition of high-performance professionals, such as athletes and firefighters. Similarly, wearable BFCs are also receiving considerable attention, both as energy harvesters and self-powered sensors^19–22^. As energy harvesters, wearable BFCs collect energy from glucose or lactate contained in bodily fluids to power small devices. Wearable BFCs as self-powered sensors utilize the fact that the power collected from glucose or lactate at any time depends on the concentration of the respective fuel. Self-powered sensors do not require an energy source for the sensing device. Some examples of wearable biosensors and BFCs are integrated into the nose-pad of eyeglasses^23^, microfluidics fabricated from a soft material^24,25^, fabricated on thin flexible film^25,26^, tattoo-type^27^, textile-based^28,29^, and paper-based^30–32^.

Paper-based devices also integrate the wicking effect of paper and can work with small sample volumes. del Torno-de Román et al. utilized paper as a fuel delivery system and achieved a power density of up to 37.5 μW cm^−2^ with 5 mM glucose^33^. Lau et al. used filter paper for fuel delivery and carbon fiber or carbon nanotube paper for the bioelectrodes and achieved a power density of 35.5 μW cm^−2^ with cascade-type 4-electron oxidation of ethanol and 26.9 μW cm^−2^ with formate, formaldehyde, and methanol as fuel and three cascade enzymes^34^. Rewatkar et al. also used filter paper for fuel delivery and multiwall carbon nanotube paper for the bioelectrodes and achieved a power density of 46.4 μW cm^−2^ with 30 mM glucose as fuel in a 4-cell-series configuration^35^.

Our group has developed several BFCs with electrodes directly printed on Japanese paper. Using Ketjenblack as electrode material and glucose oxidase as anode-enzyme, we achieved a power density of 0.12 mW cm^−2^^36^. Using MgOC as electrode material and lactate oxidase (LOx) as an enzyme, we achieved a power density of 0.113 mW cm^−2^^31^. Using GDH as an enzyme and improving immobilization, we achieved a power density of 0.12 mW cm^−2^^32^. These studies focused mainly on the anode performance. However, with a high-performing anode, the focus needs to shift to improving the cathode, especially in the case of self-powered biosensors, which need to be anode-limited in their performance.

A popular enzyme for constructing biocathode is bilirubin oxidase (BOD). One advantage of this enzyme is its capability for direct electron transfer (DET)^37–39^. As with all DET-type enzyme electrodes, the orientation of the enzyme on the electrode surface is crucial. Compared to a flat surface, a mesoporous surface structure increases the chances of the active site of a randomly oriented enzyme being within DET distance^40^; a directed orientation would increase the performance of a DET-type biocathode. Lalaoui et al. achieved an ordered immobilization of BOD on carbon nanotubes by utilizing protoporphyrin IX as a “guide” for binding the enzyme^41^. Al-Lolage et al. engineered BOD to have cysteine at a specific site and used that cysteine for a directed, covalent immobilization^42^.

In this study, we used two approaches for improving the performance of screen-printed, paper-based biofuel cells, especially the biocathode. We considered the addition of carboxymethyl cellulose (CMC) as a dispersant to the MgOC ink and investigated its rheological effect. Focusing on the biocathode, we considered bilirubin as a “guide” for immobilizing BOD in an oriented manner.

## Materials and methods

### Materials

The following materials were used in the experiment: MgOC with different average pore sizes (CNovel™, Toyo Tanso, Japan; Note: Supplementary Material Fig. S1), polyvinylidene difluoride hexafluoropropylene copolymer (PVdF; KF polymer L#9305, 5% in NMP, Kureha Corporation Japan), 1-methylpyrrolidin-2-one (NMP, Wako Pure Chemical Industries, Japan), CMC (SLD-F1, Nippon Paper Industries, Japan), Japanese paper (Izumo Tokusengasenshi, Japan), water-repellent agent (Hajikkusu, Komensu, Japan), carbon ink (JELCOM CH-10, Jujo Chemicals, Japan), 1,2-Naphthoquinone (1,2-NQ, Kanto Chemical, Japan), BOD from *Myrothecium verrucaria* (BO “Amano” 3, Amano Enzyme Inc., Japan), and LOx, which was derived from *Enterococcus faecium* and recombinantly prepared as reported previously^14^.

All other chemicals were of analytical grade.

### MgO-templated porous carbon ink

MgOC ink was prepared by dispersing MgOC and PVdF (binder; 5–6 mL/1 g MgOC) in NMP (solvent; 2.5 mL/1 g MgOC) until a smooth paste was obtained. For ink containing CMC, PVdF, NMP, and CMC (0.027 g/1 g MgOC) were pre-mixed thoroughly prior to adding the MgOC.

### Screen-printing of paper-based biofuel cell electrodes

Electrodes for the paper-based biofuel cell were fabricated similar to a previously reported method^31^. Japanese paper was treated with a water-repellent agent and allowed to dry at room temperature for 12 h. Next, current collectors were screen-printed in 5 layers using carbon ink with an LS-150TV screen-printer (Newlong Seimitsu Kogyo Co. Ltd., Tokyo, Japan) and dried at 120 °C for 12 h. The current collectors for biocathodes had 100 holes with a 0.5 mm diameter to facilitate oxygen supply^31^. Finally, 2 layers were printed using MgOC ink to form the electrodes, which were allowed to dry at room temperature for 2 d. The electrode size was 2.0 × 0.5 cm for both the bioanode and biocathode.

### BFC preparation

Electrodes were modified to form bioanodes and biocathodes similar to a previously reported method^31^. After treating with UV ozone for 15 min, the bioanode was modified by applying 20 μL 100 mM 1,2-NQ in acetonitrile and dried for 1 h. 20 μL containing 40 U LOx in 10 mM phosphate buffer was applied and the electrode was dried under reduced pressure for 1.5 h. After UV ozone treatment for 15 min, the biocathode was modified by applying 20 μL containing 5 U BOD in 10 mM phosphate buffer and dried for 1.5 h under reduced pressure. If indicated, prior to modification with BOD, 20 μL of a 0–20 mM bilirubin solution in 20 mM NaOH was applied to the electrode and dried for 1.5 h under reduced pressure; NaOH was needed to dissolve bilirubin.

### Rheological measurements

Strain dispersion of the MgOC inks was evaluated using a rheometer (MCR 102, Anton Paar, Japan) at an angular frequency of 1.0 rad s^−1^, a shear strain range of 10^–5^–10%, and a temperature of 25 °C.

### Electrochemical evaluation

The bioanode and biocathode were evaluated individually in three-electrode systems with a platinum wire as a counter electrode and an Ag/AgCl/saturated KCl electrode as reference. Cyclic voltammetry was performed with 1 M phosphate buffer as an electrolyte that contains 100 mM lactate for the bioanode. The scan rate was 10 mV s^−1^ and the potential range  0.5–0.7 V for the bioanode and 0.7– 0.2 V for the biocathode. Chronoamperometry was performed at an operating potential of 0.3 V with a measuring time of 2000 s. Biofuel cells were evaluated by linear sweep voltammetry in a controlled environment with a temperature of 36 °C and a humidity of 70%.

## Results and discussion

### CMC as dispersant in MgOC inks

**Figure 1:**
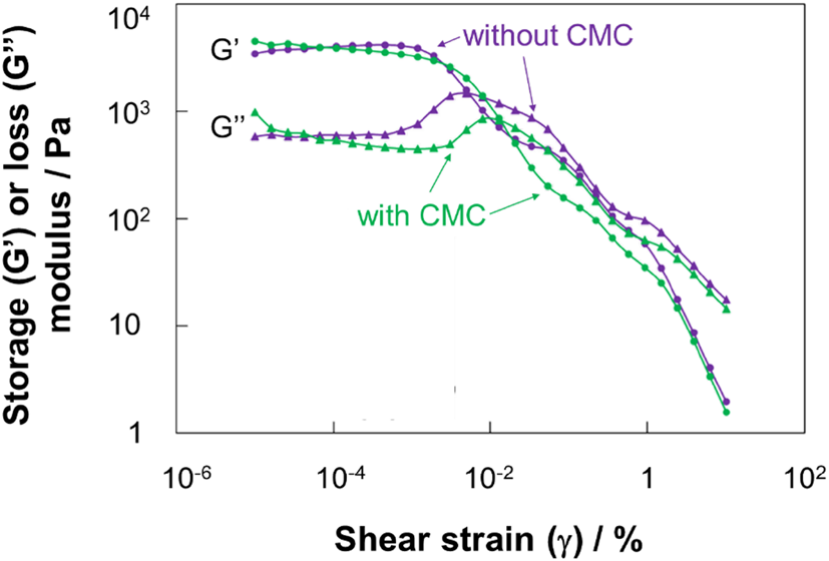
Storage and loss modulus of MgOC inks with or without CMC as a function of shear strain. Angular frequency: 1.0 rad s^−1^; temperature 25 °C. Circles: storage modulus (G’); triangles: loss modulus (G’’); green: MgOC ink containing CMC; violet: MgOC ink without CMC.

To investigate CMC as an additive for MgOC inks, the viscoelastic properties of inks with and without CMC were characterized by applying shear stress (Fig. 1). When CMC was added to the ink, the crossover point of the storage and loss moduli shifted to a higher shear strain value (7.9 × 10^–3^% without CMC and 2.1 × 10^–2^% with CMC; Fig. 1). The storage modulus represents the elastic component of the viscoelasticity, while the loss modulus represents the viscous component. Therefore, the results show that both inks are viscous at low shear strains and become more fluid at high shear strains (Fig. 1). Ink containing CMC was more stable at higher shear stress, indicating an improved dispersion (Fig. 1). Because ink is exposed to shear stress during the printing process, these characteristics are beneficial for screen-printing and should lead to more uniform electrodes.

**Figure 2:**
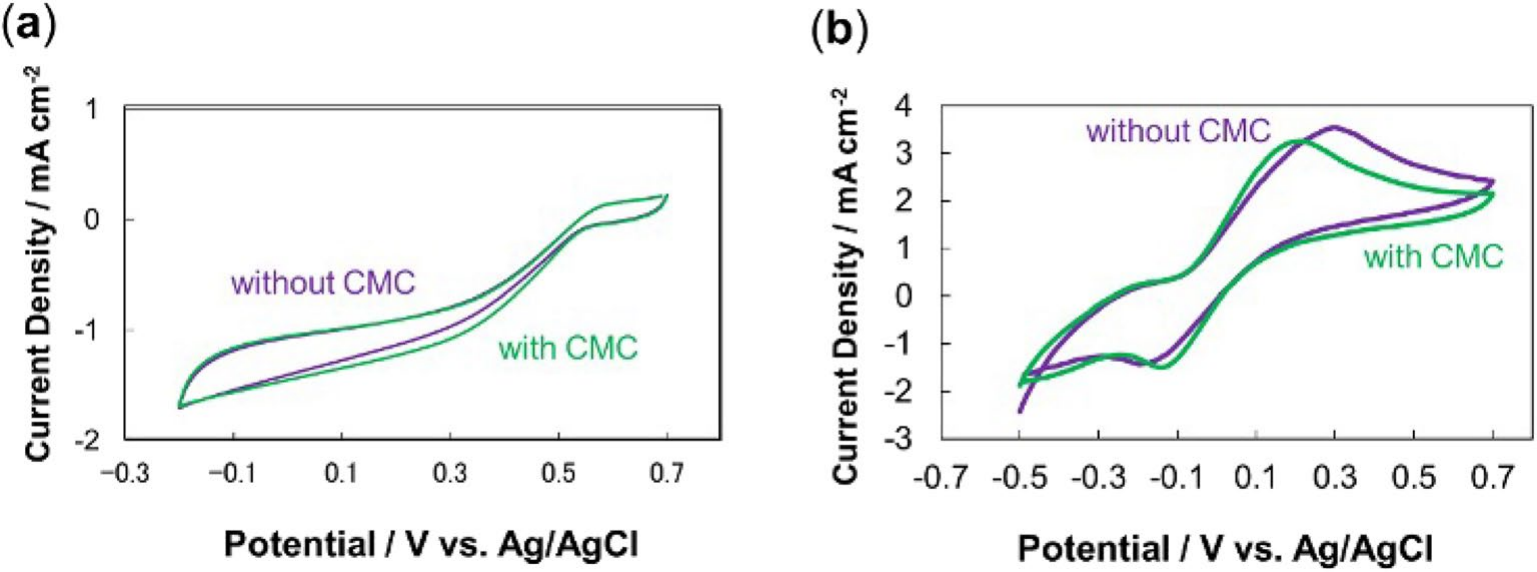
Cyclic voltammograms of (**a**) biocathodes, and (**b**) bioanodes, fabricated using MgOC inks with and without CMC. Scan rate: 10 mV s^−1^; 1 M phosphate buffer, pH 7.0; (**b**) 100 mM lactate. Biocathode enzyme: BOD; bioanode enzyme: LOx; bioanode mediator 1,2-NQ. Green: with CMC; violet: without CMC.

**Figure 3:**
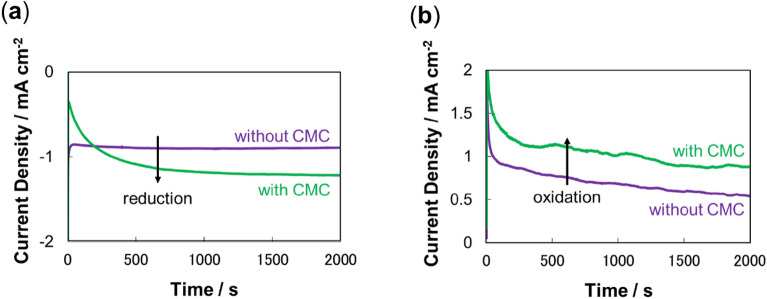
Electrochemical evaluation of (**a**) biocathodes, and (**b**) bioanodes fabricated using MgOC inks with and without CMC. 1 M phosphate buffer, pH 7.0; 0.3 V vs Ag/AgCl/sat. KCl; room temperature. (**b**) 100 mM lactate. (**a**) BOD; (**b**) LOx, 1,2-NQ. Green: with CMC; violet: without CMC.

**Figure 4:**
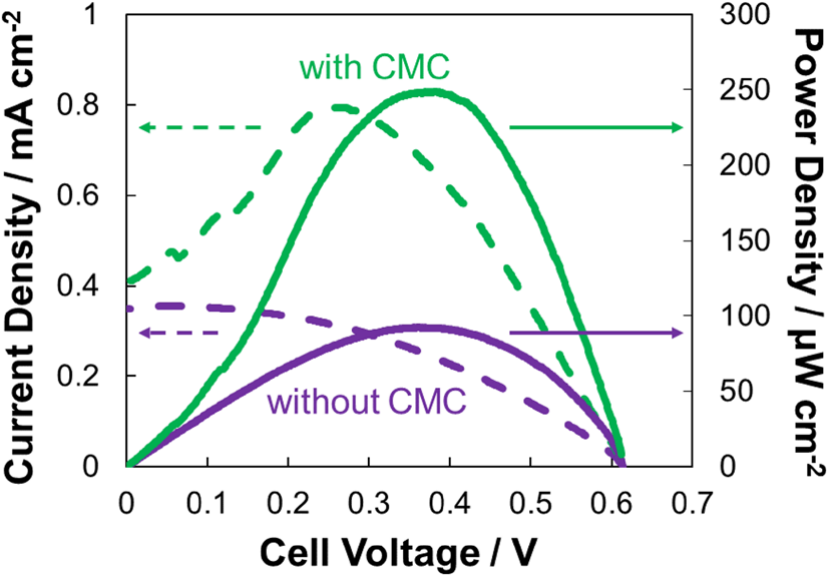
Electrochemical evaluation of biofuel cells fabricated using MgOC inks with and without CMC. 1 M phosphate buffer, pH 7.0; 100 mM lactate; humidity 70%; temperature 36 ºC. Biocathode enzyme: BOD; bioanode enzyme: LOx; bioanode mediator 1,2-NQ. Green: with CMC; violet: without CMC; straight line: power density; dashed line: current density.

Next, the MgOC inks with and without CMC were printed into electrodes, which were fabricated, modified, and characterized electrochemically (Figs. 2, 3, 4). Cyclic voltammograms of the individual biocathodes and bioanodes showed a slightly narrower peak separation when electrodes printed with MgOC ink containing CMC were used (Fig. 2). Chronoamperometric measurements showed a clear increased reduction and oxidation current for the biocathode and bioanode, respectively (Fig. 3). These results suggest that adding CMC to the ink reduces the energy needed to drive the reaction (energies in absolute terms in the reduction and oxidation directions, respectively, for the biocathode and bioanode). The similar cyclic voltammetry currents suggest that the response currents are similar in the absence or presence of CMC, when sufficient energy to drive the reaction is applied (Fig. 2). The narrower peak separation suggests that the reaction proceeds at full capacity at lower energy when CMC is added to the ink (Fig. 2). The chronoamperometric results confirm this notion: moderate energy applied at 0.3 V versus Ag/AgCl/sat. KCl seems to lead to turnover at full capacity when CMC is added to the ink, while it is insufficient to do so without CMC (Fig. 3).

When combined into a biofuel cell, the maximal current density doubled from 0.35 mA/cm^2^ when electrodes were fabricated without CMC to 0.79 mA cm^−2^ when fabricated with CMC (Fig. 4). The maximal power density increased more than 2.5-fold from 92 to 249 μW cm^−2^ by adding CMC to the MgOC ink.

Although the rheological measurements indicated that adding CMC to the MgOC ink might be beneficial for screen-printing, the difference does not explain this drastic increase in performance. Furthermore, CMC is highly hygroscopic and can improve enzyme stability^43^. The hygroscopicity can facilitate the supply of fuel to the enzyme and the stabilizing effect might prevent the loss of enzyme activity during the immobilization process. Both these properties have the potential to increase the performance of the resulting enzyme electrodes and can explain the drastic increase in the performance of the biofuel cell.

The immobilization process used in this study involves a drying step under reduced pressure. Such a procedure can lead to dehydration of the enzyme and thus to denaturation and a loss of activity^44–46^. Carbohydrates are known to prevent denaturing due to dehydration by retaining some water molecules and replacing the protein-water hydrogen-bonds with protein-carbohydrate hydrogen bonds^44–46^. Therefore, the presence of CMC in the ink might lead to more enzyme molecules remaining active on the carbon surface. Denatured enzyme on the electrode surface can interfere with the electron transfer efficiency between enzyme and electrode or mediator and electrode, which could increase the energy needed to drive the electrode reaction.

Hygroscopicity, in particular, seems to have a peculiar effect on the bioelectrode performance. A hygroscopic additive enables water to access the electrode pores during both the immobilization process, transporting more enzyme deeper into the pores of the electrode, and during operation of the device, where water is essential for the enzyme reaction, while it transports fuel deeper into the pores in the case of the bioanode. The increased supply of water to the pores might be a reason for the lower energy needed to drive the reaction, as observed electrochemically (Figs. 2, 3, 4). However, the biocathode performs more poorly when CMC is used as binder rather than an additive compared to biocathodes fabricated with hydrophobic PVdF as the binder (Supplementary Material Fig. S2), which suggests that hydrophobicity is necessary for performance and is likely ascribable to higher oxygen supply.

### Directed BOD immobilization using bilirubin

To further improve the performance of the biocathode, BOD was immobilized on the MgOC in an orientation favorable for DET. During DET, the electrode takes over the role of bilirubin in providing electrons. Thus, an orientation wherein the bilirubin-binding site of BOD faces the electrode should be favorable for DET. To achieve this orientation, bilirubin was first immobilized on MgOC, followed by BOD. The following possible mechanisms are associated with BOD immobilization: (a) BOD does not bind to bilirubin or binds in an unfavorable orientation; (b) BOD binds equally to bilirubin and to the MgOC surface; and (c) BOD binds preferably to bilirubin with its bilirubin-binding site. The resulting electrode will exhibit inferior performance compared to an electrode devoid of bilirubin when case (a) dominates. On the other hand, bilirubin is not expected to influence the performance when case (b) dominates, while the resulting electrode is expected to exhibit improved performane if case (c) dominates. Conseivable ways in which bilirubin is involved in the electrochemical mechanism include: bilirubin acting as an insulator, which is expected to decrease electrode performance, and bilirubin acting as a mediator. While the latter might improve the electrode performance, biliverdin is hardly reduced to bilirubin at carbon-based electrodes^47^; hence, this scenario is unlikely. Consequently, if improved electrode performance is observed in the presence of bilirubin then bilirubin most likely acts as a BOD immobilization “guide”, leading to the bilirubin-binding site facing the MgOC surface.

**Figure 5:**
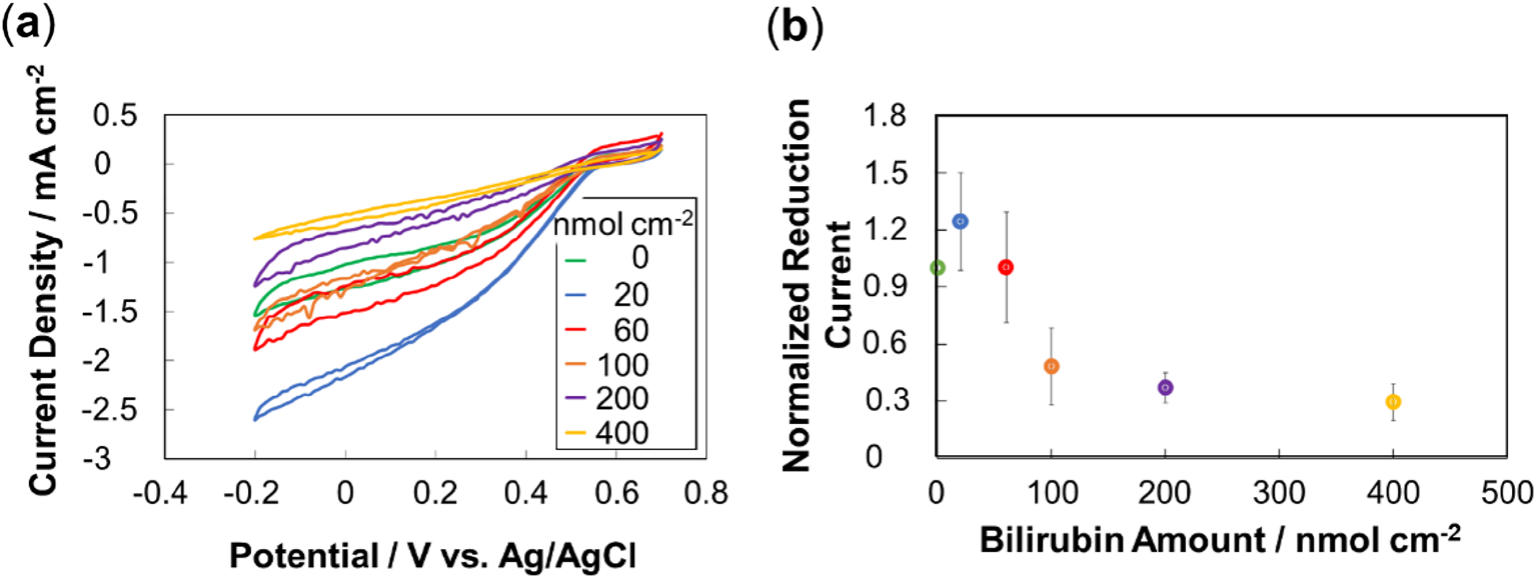
Electrochemical evaluation of biocathodes with BOD adsorbed on MgOC with bilirubin as guide for orientation. CE = Pt wire; RE = Ag/AgCl/sat. KCl; 1 M phosphate buffer, pH 7.0. (**a**) Cyclic voltammograms. Legend: bilirubin amount. (**b**) Normalized reduction current obtained chronoamperometrically vs. bilirubin amount used for immobilizing BOD. 0.3 V vs. Ag/AgCl/sat. KCl. Current for electrode with BOD immobilized in absence of bilirubin = 1.

Biocathodes were fabricated with different amounts of bilirubin as a guide for the BOD immobilization and characterized electrochemically (Fig. 5). Both the cyclic voltammetric and chronoamperometric results show that a small amount of bilirubin on the electrode leads to an increased reduction current, while a large amount leads to a reduced reduction current. A small amount of bilirubin acts successfully as a guide and helps the bilirubin-binding site of the BOD to face the MgOC surface. A large amount, however, seems to inhibit the ability of the electrode to provide electrons to the enzyme, possibly by acting as an insulating layer, confirming that bilirubin is unlikely to exhibit mediator behavior. The optimal amount of bilirubin as a guide for BOD immobilization was 20 nmol cm^−2^ (Fig. 5).

### Characterization of lactate biofuel cell including improved biocathode

**Figure 6:**
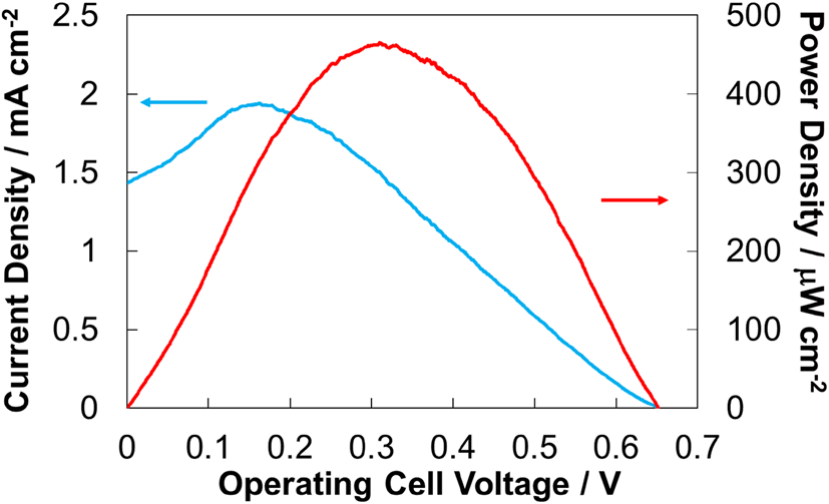
Electrochemically evaluating biofuel cells fabricated using MgOC inks with CMC and bilirubin as guides for BOD immobilization. Conditions: 1 M phosphate buffer, pH 7.0; 100 mM lactate; humidity 70%; temperature 36 ºC. Biocathode enzyme: BOD; bioanode enzyme: LOx; bioanode mediator: 1,2-NQ. Red: power density; blue: current density.

The optimized biocathode, which was fabricated using MgOC ink containing CMC and BOD and immobilized with bilirubin as a guide, was combined with a bioanode, which was fabricated using MgOC ink containing CMC with 1,2-NQ as mediator and LOx as an enzyme. The resulting biofuel cell showed an open circuit potential (OCP) of 0.65 V, a maximal current density (J_max_) of 1.94 mA cm^−2^, and a maximal power density (P_max_) of 465 μW cm^−2^ (Fig. 6). These values indicate that the BFC fabricated here is among the best performing BFCs using lactate as fuel (Table 1). An output power density approximately half that of the as-prepared value was observed in the first 24 h when stored under ambient conditions at room temperature without exposure to fuel solution (Supplementary Material, Fig. S3). After the first day, however, the BFCs were extremely stable for at least another three days (Fig. S3). Enzymes immobilized on porous electrodes are generally divided into two groups: (1) enzymes immobilized inside pores and (2) enzymes immobilized on the outer surface of the electrode. The drastic decrease in the first day is likely due to inactivation of the enzyme immobilized on the outer surface of the electrode, as the storage conditions are ill-suited to maintaining enzyme activity. The high stability from the second day onwards suggests that a significant amount of the enzyme is greatly stabilized by immobilization inside the mesopores of the MgOC. Carbohydrates are generally known to stabilize dried enzymes and are therefore often used in this capacity in lyophilized preparations and (commercial) enzyme sensor strips, and, as stated above, CMC can also improve enzyme stability^43^. Therefore, it is possible that CMC contributes to the increased stabilities of enzymes immobilized inside the mesopores of the MgOC.

**Table 1:** Performance of lactate BFCs.

BFC Type	Anode^a,b^	Cathode^a^	OCP V	*J*_max_ mA cm^-2^	*P*_max_ μW cm^-2^	Ref
Tattoo-type	LOx/TTF	Pt	0.50	n.a	44	^48^
Carbon paper	LDH/MB	LOx/hemin	0.79	0.76	380	^49^
Carbon paper/flow	LOx/FcMe_2_-LPEI	Lc/An	0.73	1.920	364	^50^
Textile-based	LOx/1,4-NQ	Ag	0.49	0.91	252	^29^
Paper-based	LOx/1,2-NQ	BOD	0.597	n.a	113	^31^
Paper-based	LOx/1,2-NQ	BOD	0.65	1.94	465	This work

n.a.: not available; a enzymes: BOD: bilirubin oxidase; Lc: laccase; LDH: lactate dehydrogenase; LOx: lactate oxidase; b mediators: 1,2-NQ: 1,2-naphthoquinone; 1,4-NQ: 1,4-naphthoquinone; An: anthracene; FcMe2-LPEI: dimethylferrocene-modified linear polyethyleneimine; MB: methylene blue; TTF: tetrathiafulvalene.

## Conclusion

In this study, the performance of a paper-based, screen-printed BFC was improved in two steps. First, the dispersibility of MgOC ink was improved by adding a small amount of CMC. The increased dispersibility was confirmed rheometrically. Thus, the fabricated BFCs showed an increased performance owing to better accessibility of the mesopores of the MgOC, as well as the stabilizing effect of CMC on enzymes. This stabilizing effect was also seen in the storage stability of the BFCs. Second, BOD was immobilized in an oriented manner using bilirubin as a guide. The resulting BFC showed an OCP of 0.65 V, a J_max_ of 1.94 mA cm^−2^, and a P_max_ of 465 μW cm^−2^, which is among the highest performance values reported to date for BFCs utilizing lactate as fuel. Although this study utilized lactate as fuel, LOx as anode enzyme, and 1,2-NQ as anode mediator, all improvements achieved should apply to other anode enzymes, mediators, and fuels.

## Supplementary Information

Supplementary Information.

## Data Availability

The data that support the findings of this study are available from the authors on reasonable request.
