# Supplementary material for: High-performance paper-based biocathode fabricated by screen-printing an improved mesoporous carbon ink and by oriented immobilization of bilirubin oxidase
Source: Sci Rep. 2022 Aug 27;12:14649. doi: 10.1038/s41598-022-19052-4 (PMC9420125; doi:10.1038/s41598-022-19052-4)
Supplement: Supplementary file 1 — Supplementary Information. [file 41598_2022_19052_MOESM1_ESM.docx]

**Supplemental Material**

**High-Performance Paper-based Biocathode fabricated by Screen-printing an improved Mesoporous Carbon Ink and by Oriented Immobilization of Bilirubin Oxidase**

Noya Loew^1,‡^, Isao Shitanda^1,2,‡,*^, Himeka Goto^1^, Hikari Watanabe^1^, Tsutomu Mikawa^3^, Seiya Tsujimura^2,4^, and Masayuki Itagaki^1,2^

^1^ Department of Pure and Applied Chemistry, Faculty of Science and Technology, Tokyo

University of Science, 2641 Yamazaki, Noda, Chiba 278-8510, Japan.

^2^ Research Institute for Science and Technology, Tokyo University of Science, 2641 Yamazaki, Noda, Chiba 278-8510, Japan

^3^ RIKEN Center for Biosystems Dynamics Research, 1-7-22 Suehirocho, Tsurumiku,

Yokohama, Kanagawa 230-0045, Japan

^4^ Division of Materials Sciences, Faculty of Pure and Applied Sciences, University of

Tsukuba,1-1-1Tennodai, Tsukuba, Ibaraki, 305-8573, Japan

^*^ Corresponding author: shitanda@rs.tus.ac.jp, ‡: N. L and I. S. are equal contributors.

**S1**


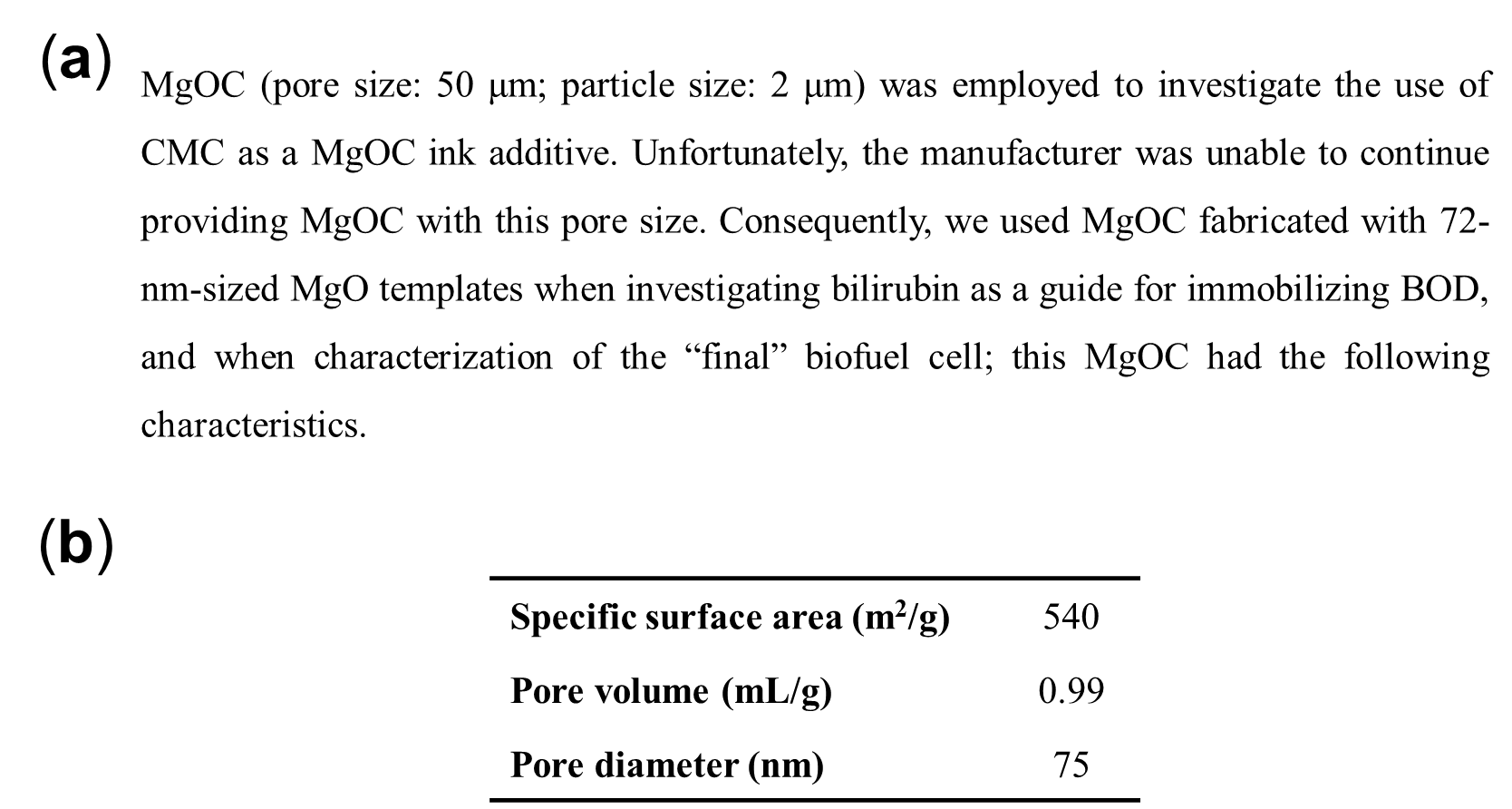


**Fig. S1.** (**a**) Note concerning pore size and (**b**) characteristic parameters of MgOC used in this study.

**S2**

**Fig. S2.** Chronoamperogram of a biocathode in which CMC was used as binder instead of PVdF for the MgOC electrode. Enzyme: BOD. Evaluation conditions: 1 M phosphate buffer, pH 7.0; 0.3 V vs. Ag/AgCl/sat. KCl.

**S3**


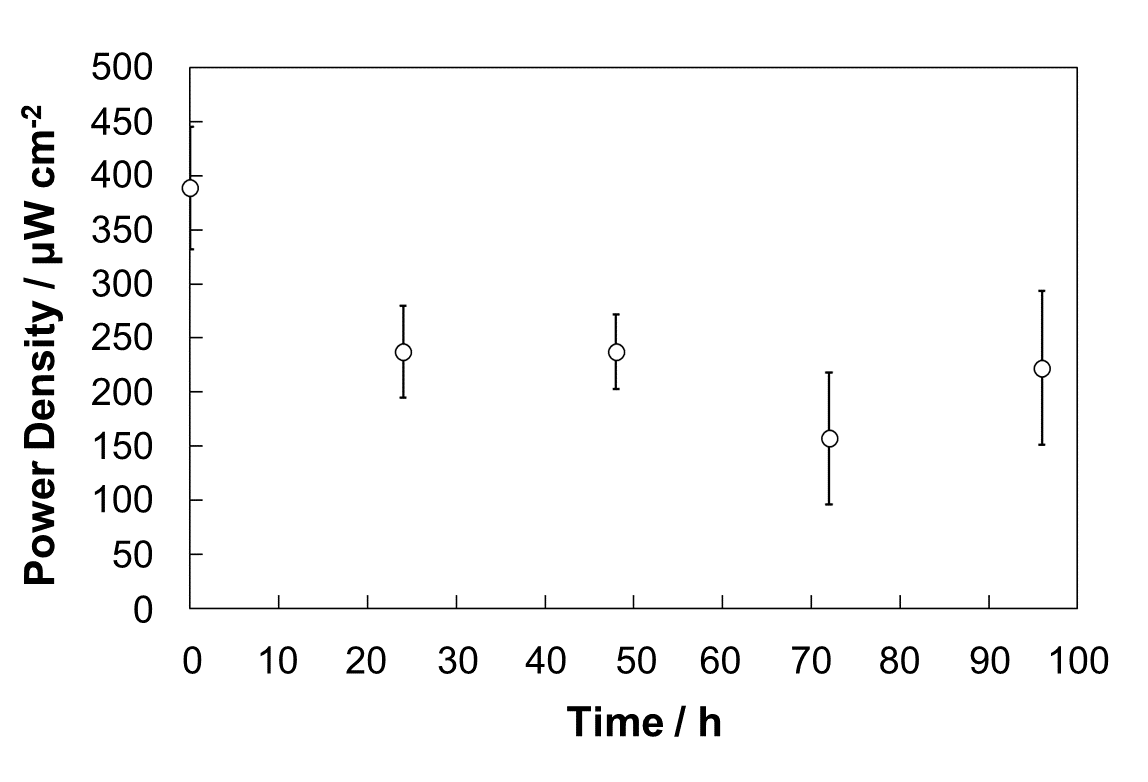


**Fig. S3.** Storage stability of BFCs. Power density of biofuel cell fabricated using MgOC inks with CMC and bilirubin as guide for BOD immobilization and stored for 24, 48, 72, or 96 h. N = 3. Storage conditions: room temperature; ambient humidity. Evaluation conditions: 1 M phosphate buffer, pH 7.0; 100 mM lactate; humidity 70%; temperature 36 ºC. Biocathode enzyme: BOD; bioanode enzyme: LOx; bioanode mediator: 1,2-NQ.
